# Content analysis of 4 to 8 year-old children's dream reports

**DOI:** 10.3389/fpsyg.2015.00534

**Published:** 2015-04-30

**Authors:** Piroska Sándor, Sára Szakadát, Katinka Kertész, Róbert Bódizs

**Affiliations:** ^1^Institute of Behavioral Sciences, Semmelweis UniversityBudapest, Hungary; ^2^Department of General Psychology, Pázmány Péter Catholic UniversityBudapest, Hungary

**Keywords:** children's dreams, development, dream research, content analysis, dream characteristics, dream interview, active self-representation, sleep mentation

## Abstract

The role of dreaming in childhood and in adulthood are still equally enigmatic fields yet to be fully explored. However, while there is a consensus at least about the typical content and formal characteristics of adult dream reports, these features are still a matter of debate in the case of young children. Longitudinal developmental laboratory studies concluded that preschoolers' dreams usually depict static images about mostly animals and body states of the dreamer but they basically lack the active representation of the self, human characters, social interactions, dream emotions and motion imagery. Due to methodological arguments these results became the reference points in the literature of developmental dream research, in spite of the significantly different results of numerous recent and relevant studies using extra-laboratory settings. This study aims to establish a methodologically well-controlled and valid way to collect children's dreams for a representative period of time in a familiar home setting to serve as a comparison to the laboratory method. Pre trained parents acted as interviewers in the course of a 6 week-period of dream collection upon morning awakenings. Our results suggest that even preschoolers are likely to represent their own self in an active role (70%) in their mostly kinematic (82%) dream narratives. Their dream reports contain more human, than animal characters (70 and 7% of all dream characters respectively), and social interactions, self-initiated actions, and emotions are usual part of these dreams. These results are rather similar to those of recent extra-laboratory studies, suggesting that methodological issues may strongly interfere with research outcomes especially in the case of preschoolers' dream narratives. We suggest that nighttime awakenings in the laboratory setting could be crucial in understanding the contradictory results of dream studies in case of young children.

## Introduction

Cortical activation during sleep and/or REM sleep-like processes are associated with vivid oneiric experiences in adults and in verbal-aged children. Since active/REM sleep has a defined developmental pattern from fetal age to adulthood, some authors assume that the case is similar with dreaming as well (Staunton, 2001). Others assume that dreaming is a cognitive achievement dependent on the maturation of the visuospatial fields of the brain, thus the formation of dreams is impossible for children until approximately 2 years of age because of their underdeveloped visuospatial skills (Foulkes, 1982, 1999). In fact, the formation and developmental processes of human dreaming are still unknown in contrast to the inspiring results from adult dream research that associate dreaming with emotional and cognitive architecture as well as cortico-cubcortical connectivity (Maquet et al., 1996; Domhoff, 2001; Pierre Maquet et al., 2005; Nielsen and Levin, 2007; Levin and Nielsen, 2009).

The first and only systematic longitudinal study of children's dreaming was done in the 1970's by David Foulkes, whose results still dominate today's developmental dream literature (Burnham and Conte, 2010). Foulkes carried out a longitudinal study (Foulkes, 1982, 1999) (children from 3 to15 years) and several cross sectional ones with various age groups (Foulkes, 1967, 1979; Foulkes et al., 1967, 1969, 1990). The majority of these studies used a laboratory based stetting of dream collection with EEG monitoring and systematic nocturnal awakenings requiring immediate dream reports to the laboratory assistant personally or via intercom.

Foulkes' results provided a surprising alternative to the general opinion about young children's dreams considered to be relatively vivid, colorful and creative with strong feelings, especially negative ones appearing in form of frequent nightmares (Kimmins, 1920; Foster and Anderson, 1936; Despert, 1949), In his study 3 to 5 year-olds reported infrequent (17% of REM awakenings) and brief (average 14 words) dream reports, that usually lacked movements and actions (static imagery), emotions, an active self-character and a clear storyline. Moreover, human characters and interactions rarely appeared in the reports, instead children frequently dreamt about body-state themes, especially those relating to the sleeping self, and about animals. Thus, typical dreams of this age were “I was sleeping in the bathtub” or “A fish in a bowl on the riverside.”

Foulkes first observed kinematic imagery and social interactions in dreams between 5 and 7 years. In the laboratory studies it was only between the ages of 7 and 8, that the children's reports not only got more frequent (43%) and significantly longer with more complex narrative structure, but active self-representation together with thoughts (10% of all reports) and feelings also appeared. It was only between the ages of 9 and 11 that dream report frequency reached a median of 79%, close to the typical adult REM dream reporting frequency (85–90%), with report frequency and length becoming stable individual parameters of each child. Gender differences also seemed to appear first from the age of 7 years manifesting in the percentage of male and female characters (girls dreamt about more female characters than boys) and differences in the frequency of activities (boys tended to perform more gross-motor activities) (Foulkes, 1982) in a statistically significant manner. Although, Foulkes (1982) reported gender differences in the number of male strangers (higher in boys' dreams) and social approach initiations (higher in girls' dreams), as well as a reanalysis of his results found differences in the male/female character percent (coded according to the Hall/Van de Castle system, see Domhoff, 1996), both already present from 5 years of age, none of these latter results claim statistical significance due to inadequate sample size.

Interestingly, studies using non-laboratory-based setting and a different methodology yielded strikingly different results on most aspects of preschoolers dreams (Sándor et al., 2014) although results tend to become more convergent with age. Most importantly Strauch and Meyer found similar results to those of Foulkes' in their laboratory-based study with children aged 9–15 years and agreed that “dreaming is, for the most part, adult-like, by the ages of 9–11 years” (Strauch, 2005, p. 166).

A typical home setting involves one of the parents carrying out a structured dream interview with the child upon morning awakenings. Two studies conducted in the home setting, that included the age range between 3and 5 years, yielded somewhat different results from those of the laboratory studies. For instance in Colace's (2010) study the dreams tended to be longer than those collected under laboratory conditions (mean world count: 35 words). Resnick et al. (1994) using morning interviews found no difference in dream recall frequency between the 4 to 5 and 8 to 10 year age groups (56 and 57%, respectively). These results are in line with the nursery/kindergarten-based research where the most common means of dream assessment is typically personal interviews (Beaudet, 1990; Colace, 2010; Honig and Nealis, 2012) or individual play sessions (Despert, 1949), some of which allowed for story telling parallelly to dream reports in order to avoid social pressure in favor of reporting dreams (Honig and Nealis, 2012). The only parent-recorded questionnaire-based study using a wide age group (from 2 to 16 years) showed that most of the parents rated their preschool aged children's dream reports as being short stories (57.6%, rather than short or long sentences: 32.6%).

Home, school, and questionnaire-based studies are also consistent in reporting the ratio of active self-representation to be predominant in preschooler's dreams. Dreams including active self-representation reached 80% in both the preschooler and the school aged groups in Resnick et al. (1994) study and were predominant in preschool-based (59.4%) (Honig and Nealis, 2012) and questionnaire studies (56%) (Colace, 2006) as well.

In contrast to laboratory results human characters were shown to be common in young children's dreams at home (29% of all characters (Resnick et al., 1994), at the nursery (80%, Beaudet, 1990) and also in a questionnaire study where Colace (2006) found that family members were the most commonly appearing characters in dreams (in 60% of the dreams). Family members were found to be especially high in preschoolers dreams [main characters in 30% of the dreams (Honig and Nealis, 2012) and most of the characters in Resnick et al.'s (1994) study]. Animal characters were found to be less frequent than humans in all non-laboratory settings [13% of all characters (Resnick et al., 1994), in 43% of the dreams (Honig and Nealis, 2012) and in 49% of the dreams (Colace, 2006)].

Preschool-based studies (for example: Honig and Nealis, 2012) also pointed out that almost all of the dreams of young children depict motion and activities (81.2%), and that feelings appearing in the dreams are common (in 75% of the reports).

In contrast to Foulkes who found that social interactions were missing from preschooler's dreams, Colace found that social interactions in dreams were rather frequent (in 67.4% of all reported dreams). Oberst (Oberst et al., 2005), who asked children to write down their “last remembered dream,” also analyzed social interactions in dreams. She specifically looked at aggressive interactions, and found that 75% of the dream reports contained aggression amongst the 7 to 8 year-olds, her youngest age group. This percentage then showed a decreasing tendency until adolescence.

Interestingly, the observed gender differences in dream content and dream recall frequency (with girls reporting more dreams than boys, Strauch, 2005) seem to be relatively stable across the various studies. Most of them confirm the difference in the male—female character percent (Hall, 1984; Strauch and Lederbogen, 1999; Strauch, 2005), with some of them also pointing out more characters in the dreams of females (Saline, 1999). The majority of studies also found a difference in social interactions, agreeing that while boys report more aggression, girls experience more friendly interactions in their dreams (Strauch and Lederbogen, 1999; Oberst et al., 2005; Strauch, 2005). It is important to note that only in Honig and Nealis' study Honig and Nealis (2012) did the authors find statistically significant gender differences in the preschool age, namely in the type of activities (boys dreaming about more chasing and girls about more play) and feelings (girls reporting more joy and boys more anxiety) children's dream reports depicted. Since preschoolers' dreams did not show gender differences in the laboratory studies this is also a divergent point worth of further investigation.

The presence of emotions in children's dreams is also highly dependent on the research settings and dream collection methods used (Sándor et al., 2014). In the laboratory settings Foulkes (1982) found that only 8% of dreams contained emotions. This value grew to 10–25% in the 5 to 7 years-old. In contrast, most of the dream reports of 3 to 5-years old (Despert, 1949) or more specifically 75.9% of them (Honig and Nealis, 2012) contained at least one emotion when children were interviewed in the nursery/school.

Summarizing the divergent results in the literature we arrive to three topics that are crucial especially in connection with preschool aged children's dreams.

Firstly, interviewing children about their dreams, we have to be sure that they understand the concept of dreams. Recent studies on children's understanding of the non-physical, private, and internal nature of dreams show that children even from the age of 3–4 develop an understanding of such concepts (Woolley and Wellman, 1992; Meyer and Shore, 2001), although these results are not universal (Woolley, 1995) and other aspects of understanding, like the origin of dreams seem to stabilize only by the age of 5. Some other aspects, like beliefs about the controllability of dreams continue developing during the early elementary school years (Woolley and Boerger, 2002). These results tend to mirror the intensive cognitive maturation between the ages of 3 and 7 years (Kagan and Herschkowitz, 2005). On the whole, with counting for the great individual variability, preschoolers still could be considered as reasonably competent dream reporters, but the extent to which the gaps of their dream narratives are filled in with the products of waking fantasy is still a matter that needs to be dealt with subjectively by the researcher or the parent.

The second topic deals with the assumed cognitive influences on the maturation of dreaming. It is shown that indices of formal dream characteristics increase with age, and this also means an influence of growing cognitive abilities, for example longer dream reports might correspond to children's growing verbal and narrative abilities. There is convergence in the literature that some features of dream content, for example bizarreness, also parallel cognitive improvement (Resnick et al., 1994; Cicogna et al., 2007; Colace, 2010) together with the positive association of dream recall frequency and visuo-spatial abilities (Foulkes, 1982, 1999). It is also important to stress that though certain cognitive skills may shape the cognitive and emotional processing of the dreams and the formation of dream narratives, we do not have direct evidence to conclude that these skills determine the development of dreaming as an experience. On the other hand our findings as well as several earlier reports cited above suggest that the relationship between dreaming and cognitive-affective maturation is still an area to be discovered in more detail and it is evident that the preschool age is the most significant puzzle and controversial issue in the realm of this maturation process.

The third topic addresses the importance of methodology. Looking at the available data emerging from different sampling procedures it seems obvious that different methods suit different research objectives, but they also seem to have an effect on the outcomes. It is not accidental that the most passionate debate of dream research has long been the question of setting. Some authors found home dreams in adults and generally being more dramatic (containing more aggression, friendliness, misfortune, and good fortune) than laboratory dreams (Domhoff and Kamiya, 1964; Hall and Van de Castle, 1966; Weisz and Foulkes, 1970) which was confirmed in adolescents (Strauch, 2004). Foulkes (1979) systematically compared children's dreams under home and laboratory conditions. He reported no significant differences in home and laboratory dream recall frequencies of 3 to 4 years-old children. However, he found significant differences between dreams from the two settings in 6 to 7 years-olds: home dream reports were more likely to be unpleasant, to contain a definite setting, more fear than happiness, more antisocial than prosocial motivation, more self-directed outcomes (both favorable and unfavorable), as well as fewer words than laboratory reports. Other studies of young children's dreams conducted in the home or preschool/school settings found dream reports to be richer in motion, self-representation, human characters and interactions than the usual laboratory reports (Resnick et al., 1994; Honig and Nealis, 2012). The laboratory vs. non-laboratory differences were interpreted as recall biases in the home settings (Foulkes, 1979), or unfavorable effects of the artificial environment (Resnick et al., 1994; Honig and Nealis, 2012).

Some researchers claim that the only reliable way of investigating dreams is in the neutral laboratory environment via REM sleep awakenings and dream interviews carried out on the spot by a neutral laboratory assistant (Foulkes, 1999). Most likely the awakenings during REM sleep minimizes the chance of recall bias toward the more salient dream experiences and also the confabulatory tendencies that might fill in the gap in the storyline that are both major risks in home and school based studies. On the other hand authors expressed their doubts about the appropriateness of the laboratory environment for young children (Resnick et al., 1994; Bulkeley et al., 2005); the unknown laboratory environment and interviewer may cause disorientation or difficulties for the children talking about their dreams (Resnick et al., 1994) or even influence the dream experience itself (Domhoff, 1969). Although, Foulkes et al. (1990) reported low anxiety levels of the children during the laboratory visits, the laboratory environment might not be an everyday experience for the children. He also mentions that in his first longitudinal study, parents were not allowed to stay with the child overnight in contrast to the later cross sectional research, in which he did not include children of preschool age (3 to 5 year-olds). The reason for this is that he himself was not convinced by the validity of preschooler's dream reports, not because of the content of these reports but because of the divergent social-cognitive correlates of dream recall frequency in this age group. Dream recall frequency correlated with social and verbal skills in this youngest age group in contrast to the consistent correlation with visuo-spatial abilities in all the other age groups. A possible source of this divergence and also the barren nature of preschooler's dream reports might be that they could not be fully aroused during the interviews, thus more social children may have put more effort in reporting at least something about their mentation at the time of awakening. If this is the case it might not be the laboratory itself but the method of nighttime awakenings that is simply unsuitable for young children (Sándor et al., 2014; see for example: Foulkes and Shepherd, 1971). In case of the older age groups findings across the different methods tend to be more convergent and results of the original longitudinal laboratory studies (Foulkes, 1982, 1999) are supported with the later cross sectional ones (Foulkes et al., 1990; Foulkes, 1999) and as well as by Strauch et al. (Strauch and Meier, 1996; Strauch, 2005).

However, the non-laboratory settings have their own inherent shortcomings too. One of them is the lack of control for the sleep-waking state before the interview. Another one is the time lag between the dream experience and the dream interview which has a significant effect on the dream recall frequency and possibly the dream content as well (Koulack and Goodenough, 1976). Moreover, the risk of the dream memory interfering with waking stimuli and the penetration of waking fantasy and interpretation processes into the narrative is significantly higher in case of morning dream interviews compared to on the spot nighttime ones (Schredl et al., 2003). Also the interviewer plays an important role in dream interviews: the stability and neutrality of the research assistant in the lab are possible advantages compared to parents in home settings who might have unspoken expectations toward the child's dream life (Foulkes, 1999). Thus, it seems plausible to conclude that the divergence between laboratory and non-laboratory research findings on dreams of preschoolers find its roots in the different biases and methodological problems inherent to the above approaches.

It is surprising that considering the methodological controversies that arise, especially in the case of research on young children's dreams, there have been few attempts to clarify the picture. Now aiming to find a compromise between the two sharply differing approaches of neutrality and familiar environment we propose a home based study with well-established methodological details. Since the key to the success of laboratory study results lies in its well-controlled methodology, we aim to propose a similarly carefully thought-out and controlled method based on dream collection at home using a longer term data collection period and including 40 children.

## Aims and hypothesis

Our aim is to give a description of 4 to 8 years-old children's dream characteristics and dream content in a familiar home environment and using a reasonably neutral and controlled method of dream collection.

Here we focus on those dream characteristics that diverge most prominently across different dream collection methods. These are: human and animal characters, active self-representation, human social interactions, kinematic imagery and voluntary activities, and emotional load and dream quality. Our main theses guiding the descriptive research aspects of the study are the following:

1. Humans including the active self as opposed to animal characters are predominant in the dream plots irrespectively of the age of the dreamer.
2. The majority of the dream reports contain kinematic imagery, including self-initiated actions in the dreams.
3. Social interactions and self-reported emotions are already present in young children's dreams (4 to 5 year-olds).

Regarding gender and age the following specific hypotheses are formulated:

1. There is a gender difference in the male–female character percent in the dream reports.
2. A higher rate of aggressive content is observable in boys' dreams.
3. The frequency of aggressive interactions in dreams is characterized by an age-dependent decrease.
4. The number of cognitively reflective verbs increases with the age of the dreamer.

## Materials and methods

### Subjects

Participants were 40 children and their parents recruited from different schools and kindergartens in Budapest, Hungary, who agreed to take part in the study. The sampling method was convenience sampling and snowball sampling as this latter turned out to be the most efficient way to collect subjects for this time demanding study. The children were between the ages of 4–8.5 years (min: 3.8, max: 8.7, mean: 6.3 years, SD: 1.6), evenly sorted into three age groups: 14 children (7 females) between the ages 3.8 and 5.5 years (Group1, mean: 4.5 years, SD:.56), 12 children (7 females) between the ages of 5.51 and 7 years (Group2, mean: 6 years, SD:.36), and 14 children (7 females) between the age of 7.01 and 8.5 years (Group3, mean: 8.1 years, SD:.53).

All of the children were from a middle class, educated environment with at least one of the parents holding a degree in higher education.

All the children were healthy; any diagnosis of mental or physical illness caused an exclusion from the study. Written consent forms were obtained from the parents. Ethical approval of the study was received from the Semmelweis University Ethical Review Board.

### Methods of data collection and control

An initial interview with both the parents and the children was carried out, where they were informed about the details and schedule of the study: children could express willingness to participate and the parents could sign the written consent forms. The parents were trained how to use the structured dream interview developed for this study and how to avoid suggestive questioning.

Dreams were obtained from the children upon morning awakenings over a 6 week period of time in form of a structured dream interview conducted by the pre-trained parents (for example see Supplementary Material). The 6 week-period was considered to be long enough to provide a representative sample of the children's dreams. In order to meet the children's possible need for extra attention in the morning we asked the parents to carry out a 5 min conversation after waking about the night or any other topic that the child showed interest in, even if no dream was recollected. In that way the possible need for attention did not pressure children to make up dream reports.

Interviews were carried out within the first 20 min of the waking state each morning and were tape recorded in order to allow retrospective control over the conversation.

In order to rule out parental suggestions and waking fantasy penetrations from entering the reports, we introduced a three step control system on the dream collection and evaluation process which included control of the child's narrative by the parent and the researcher, and control of parental influence by the researcher. The steps are:

1. The parents were asked to rate the dream reports on a 0-10 scale in order to estimate the extent to which the report is a dream (10) or a product of waking fantasy (0). Dreams rated below 5 points were excluded from the research. Parents were also encouraged to name a point (if recognized) where the dream becomes a fantasy, and thus we were able to exclude the products of waking fantasy from the analysis.
2. A research assistant, blind to the parent's ratings, rated the dreams independently on a similar scale using the guidelines of Colace (Colace, 1998, 2010) on dream report credibility. Those dreams, where the two ratings diverged significantly, were excluded from the analysis.
3. Answers to suggestive questions were eliminated from the dream narrative: mentioning any concrete character or event in the parent's question that the child had not mentioned before were considered suggestive (for example: “Was your father there in the dream?” instead of the general “Was there anyone else in your dream?” or “Did anything happen in your dream?”).

### Data analysis

After the 6 weeks of data collection, assistants, blind to the purpose of the study, typed the conversations into written documents. During the word-count process two independent researchers counted relevant words of the dream reports based on the word-count rules described by Foulkes and Shepherd (1971).

To form the basis of our system we considered two popular content analysis systems, which we augmented with some of our own categories. One of these existing content analysis methods for children's dreams was developed by Foulkes and Shepherd (1971) and the other one was the widely used system of Hall and Van de Castle (1966), which we simplified to fit the characteristics of the often short and simple dream reports of children. We also modified the Foulkes-system to be comparable with the other. Here, we focus on those dream characteristics that are most prominently divergent across different dream collection methods and could be important in clearing up inconsistencies in the literature^1^.

1. Dream characters
  - Human character: a character is someone physically present in the dream, or whom the Dreamer interacts with in the dream. The dreamers themselves were not counted as human characters; they were only coded as self-representation.
  - Animal character: any kind of non-imaginary animal physically present in the dream.
  - Active self-representation: coded if the dreamer is present and actively takes part in the dream mentation (e.g.,: I was on a ship or I made a cake). Passive self-representation is scored if the dreamer merely views the scene and no self-representation is coded if the dreamer is not mentioned at all.
2. Kinematic nature of dreams
  - Kinematic imagery: children were asked if their dream contained motion (“Did you see your dream as a motion picture was it rather like a photo?”).
  - Self-initiated actions: any activity actively performed by a character is scored here. For example: “I reached for the cup…” or “I went to my grandma's place.”
3. Social interactions
  - Aggressive interactions: any hostile or offensive act towards a character, when it is a consequence of a deliberate, intentional act on the part of one character to harm or annoy some other character. For example: “I was playing with E. and she hit me on the head…” or “… the sharks hit each other with the iron rods…”
  - Friendly interactions: any friendly or helpful overture toward another character, which involve a deliberate, purposeful attempt on the part of one character to express friendliness toward or cooperate with another. For example: …”she stroke the pony…” or “The natives were took the polar bear to their hut to cure it…”
4. Cognition in dreams: mental or intellectual activity of any sort. For example thinking, planning, counting, decision making, imagination, forgetting, remembering, dreaming, learning, knowing, comparing, longing for, expectation, will, or interest. Negative examples are also scored. For example: “I did not remember the way home…”
5. Emotions in dreams and emotional dream quality
  - Dream emotions: the children were asked if they had any feelings during the dream and they were given examples of feelings by the interviewer. (“Did you have any feelings during the dream? Did you feel for example angry, sad, happy, surprised or scared or were just calm?”). Positive emotions were happy, good (sometimes they just spontaneously said they felt good) and calm. Negative emotions were sad, scared, angry and bad.
  - Emotional dream quality: is also based on the children's self-report. (“How was this dream? Was it good, bad or neutral?”)

All of the above categories were coded by two independent raters. For measuring Inter-rater reliability we used Cohen's Kappa which varied between 0.7 and 0.87 amongst the content analysis categories.

Since the number of observations per child varied greatly across the sample, dream content characteristics were relativized (item/dream) for each child. These were the units of the statistical analyses. Statistical analysis of the age-dependency of dream report features was performed by calculating the Kendall tau rank correlation coefficient. Between-group comparisons, including age categories and gender differences in children's dream content were tested with the Kruskal–Wallis test, and the Mann–Whitney *U*-test serving as *post-hoc* testing. We also report effect sizes as advised by Field (r = Z/√N) (Field, 2013).

## Results

### Dream report frequency and length

Over the 1680 attempts (42 mornings for each of the 40 children) 349 dreams were collected, with an overall mean of 8.7 dreams per child (ranging from 1 to 25 dreams). Group 1 accounted for 112 dreams with an average of 8 dreams per child. Children in Group 2 reported 129 dreams with an average of 10.8 dreams per child, and Group 3 subjects collected 108 with an average of 7.7 dreams per child. There was no significant correlation between dream report rate and age nor significant difference amongst the three groups (Kruskal–Wallis *H* = 1.09, *p* = 0.58).

**Figure 1:**
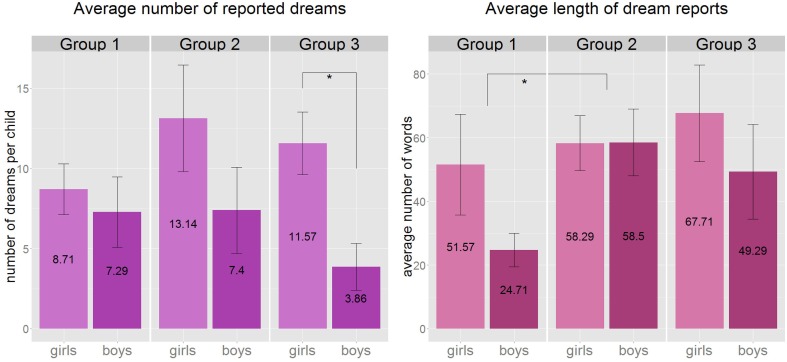
**Left:** the average number of reported dreams by gender and age group. We observed a significant difference in the number of dreams between the two genders in the oldest age group (*U* = 44, *p* = 0.01, *r* = 0.67). This gender difference was also present in the overall population. **Right**: the average length of dream reports by gender and age group. A significant increase was present between Group 1 and Group 2 in the length of dream reports (*U* = 31, *p* = 0.007, *r* = 0.53) when genders were tested together. ^*^p < 0.05, Group1: 3.8–5.5 years, Group 2: 5.51–7 years, Group 3: 7.01–8.5 years.

Gender differences were found in the overall number of reported dreams (Mann–Whitney *U* = 298, *p* = 0.008, *r* = 0.42, *df* = 38) which also appeared in the oldest age group where girls reported more dreams than boys (*U* = 44, *p* = 0.01, *r* = 0.67, *df* = 12; Figure 1). Dream recall frequency seems to be very similar in the youngest age group, but while the girls' relative dream recall shows a clear rising tendency toward the oldest age group, the boys' relative report rate declines (from 46 to 25%).

We also tested for the possible changes in dream recall rate throughout the 6 weeks of data collection. We divided the 6 weeks into three 2-week periods and found that children reported significantly more dreams during the first 2 weeks (mean dream recall: 5) of the dream collection period than either of the second (*U* = 345, *p* =.0002) or third 2-week periods (*U* = 50.5, *p* = 0.0025) which latter two showed no difference (three dream reports on average).

Dream length was measured by the number of words in the dream report (Foulkes and Shepherd, 1971). The median of the number of words across the dreams was calculated for each child. The overall mean of the median lengths of all children's dreams was 51.3 words (median: 39.5, ranging from 12 to 143 median words per child). The mean length was 38.1 words in Group 1 (median: 32.2), 58.4 words in Group 2 (median: 60.8), and 58.5 words in Group 3 (median: 43.5). We found significant differences between the dream lengths of the age groups (Kruskal–Wallis *H* = 6.04, *p* = 0.048), with the significant difference observed between Group 1 and Group 2 (Mann–Whitney *U* = 31, *p* = 0.007, *r* = 0.53, *df* = 24; Figure 1), but there was no significant correlation with age as a continuous variable. We found no significant gender-difference across the three age groups, only that in the youngest age group a tendency emerged with girls reporting longer dreams (*U* = 267, *p* = 0.069, *df* = 38) than boys.

### Dream characters

**Figure 2:**
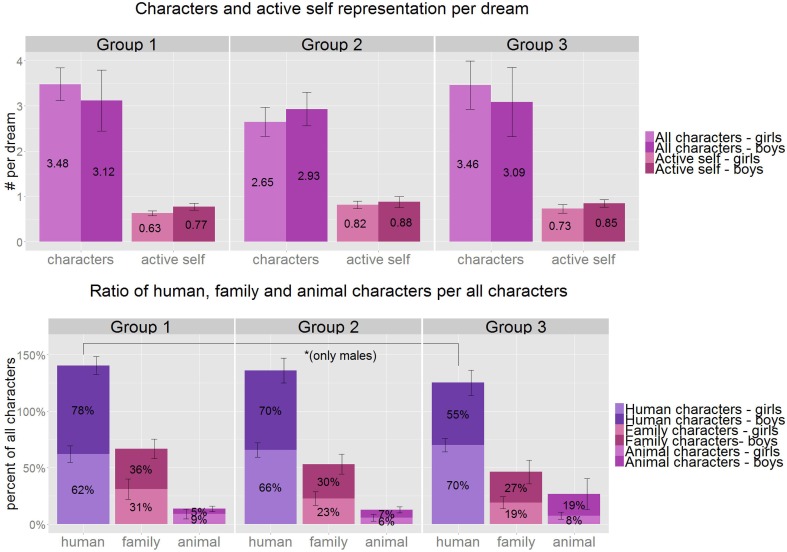
**Above:** the average number of characters per dream by gender and age group (purple). The ratio of dreams with active self- representation compared to all dreams (pink). **Below**: the percentage of human, family, and animal characters of all characters appearing in the dreams by gender and age group. Although, there is no significant age or gender difference in any of the above variables, boys show a significant decrease in the percentage of human characters in their dream reports between Group1 and Group3 (*W* = 40.5, *p* = 0.047, *r* = 0.39). ^*^p < 0.05, Group1: 3.8–5.5 years, Group 2: 5.51–7 years, Group 3: 7.01–8.5 years.

Altogether we counted 1092 characters in the 349 dreams, an average of 3.13 characters per dream (not including the self). The most frequent characters in the dreams were human characters, with an average of 2.15 per dream (749 altogether) accounting for 68.6% of all characters. Only 7.9% of the characters were animals. The average number of characters did not show a significant change across the age groups (3.3; 2.8; 3.5, respectively), nor did the percentage of human (71, 68, 68, respectively), or animal characters (9, 6, 14%, respectively) to all reported characters. We found no significant gender differences in dream characters, but boys independently from girls showed a declining pattern of the relative number of human characters between Groups 1 and 3 (*U* = 40.5, *p* = 0.047, *r* = 0.39, *df* = 12; Figure 2). We found the expected gender differences in the distribution of male and female characters: girls dreamed about more female characters (*U* = 279, *p* = 0.01, *r* = 0.40, *df* = 38), and boys dreamed about more male characters (*U* = 114, *p* = 0.035, *r* = 0.33, *df* = 38). We also calculated the male per female character percent from the Hall/Van de Castle system (Domhoff, 1999) and found a significant gender difference appearing even in the preschool age group (*U* = 76, *p* = 0.004).

#### Self-representation

The dreamer's own self appeared in an active role in 77.6% of the dreams, which did not differ significantly between the age groups. Analyzing the differences between the age groups a tendency of growth is observable between Groups 1 and 2 (*U* = 52, *p* = 0.097, *df* = 24). No gender differences were present in connection with the self in the dreams.

An example of a typical dream depicting various characters and the active self from a 5.7 year old girl: “We went to the city park, papa and you [mom] and Lili and Bende [siblings] we went for a walk and we arrived at a garage…”

### Kinematic imagery

**Figure 3:**
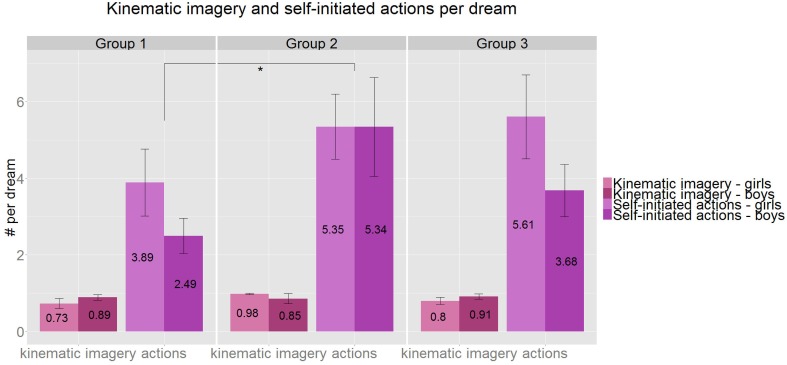
**The ratio of dreams with kinematic imagery (pink) is stable and stays high throughout the age groups (ranging from 73 to 91%)**. The average number of Self-initiated activities per dream (purple), show a significant increase between Group 1 and Group 2 (*U* = 42, *p* = 0.033, *r* = 0.34). ^*^p < 0.05, Group1: 3.8–5.5 years, Group 2: 5.51–7 years, Group 3: 7.01–8.5 years.

Eighty six percentageof all dreams were kinematic out of those dreams where kinematic or static nature was explicitly reported by the dreamer. The kinematic or static nature of the dreams was reported in 84% of all dream reports (293 dreams), and this ratio did not differ significantly between the age groups (82, 81, 89%, respectively). Also the percentage of kinematic dreams itself did not differ significantly but stayed relatively high across the age groups (80, 93, 85%, respectively). No gender differences were detected in connection with kinematic imagery in the dream reports (Figure 3).

#### Self-initiated activities

As further evidence adding to the self-reported kinematic nature of the dreams, we counted self-initiated actions in the dream reports. We counted 1651 activities in the 349 dreams altogether, which on average is 4.73 activities per dream. 90.2% of all dreams contained at least one activity.

Thus, a typical dream report of 4 to 5 year-olds is likely to be kinematic and contain more than one self-initiated actions:”… then the ship started to sink and Bius [sibling] and papa swam over to me and then deep-sea divers found us and they carried us to the dry land…” (boy, 4.9 y) or “We ate some cookies and then we went to the playground at Mammut [shopping mall]…” (boy, 4.7 y).

The number of all activities tended to increase across the age groups (3.8, 4.8, 5.8 activities per dreams respectively) (Kruskal–Wallis, *H* = 5.51, *p* = 0.063). This increase was significant between Groups 1 and 2 (*U* = 42, *p* = 0.033, *r* = 0.34, *df* = 24; Figure 3). The ratio of dreams containing activities is similarly high and stable across the age groups.

Girls reported slightly more dream activities than boys, which remained a tendency (*U* = 268, *p* = 0.065, *df* = 38).

### Social interactions

We counted altogether 321 interactions in the 349 dreams which make up an average of 0.92 interactions per dream. 57.1% of all dreams contained at least one interaction. Aggression accounted for 38.3%, friendliness for 45.8% of all interactions (**Figure 5**). Out of all the dreams 27.7% contained any kind of aggression and 35% involved friendly interactions.

The number of interactions per dream (1, 0.8, 1) and the percentage of dreams with at least one interaction (55, 54, 62%) remained stable across the age groups. Within the stable interaction rate we observed an increasing tendency of the relative number of aggressive acts per all interactions with age (tau = 0.21, *p* = 0.06, *df* = 38). The percentageof aggressive interactions relative to all interactions was characterized by an intergroup increase (Kruskal–Wallis, *H* = 6.39, *p* = 0.04) from Group 1 to Group 2, as well as from Group 1 to Group 3 values (*U* = 41, *p* = 0.029, *r* = 0.43, *df* = 24 and *U* = 52, *p* = 0.034, *r* = 0.40, *df* = 26, respectively). The number of dreams with at least one aggression per dream also showed an increase between Group 1 and 3 supporting the above results (*W* = 54, *p* = 0.043, *r* = 0.39, *df* = 26, **Figure 5**).

Examples of aggressive interactions typically varied on a wide scale from mild sibling arguments: “I took the hammer from her hand and she started crying…” (girl, 4.2 y) to deadly actions: “the car ran over me…” (girl, 3.8 y).

A decrease in friendliness per all interactions (Kruskal–Wallis, *H* = 10.4, *p* = 0.005) was also detected, which is significant between Group 1 and 2 and Group 1 and 3 (Mann–Whitney *U* = 29, *p* = 0.005, *r* = 0.55, *df* = 24 and *U* = 41, *p* = 0.009, *r* = 0.50, *df* = 26 respectively), yielding a tendentious negative correlation with age (tau = 0.21, *p* = 0.059, *df* = 38).

Friendly interactions usually included giving or accepting help or playing/doing mischief together: “… we found out that we will escape from school together … and we climbed over the fence and … went to the amusement park” (girl, 7.6 y) or “ I dreamed that mother was telling me a good night tale” (girl, 5.2 y).

**Figure 4:**
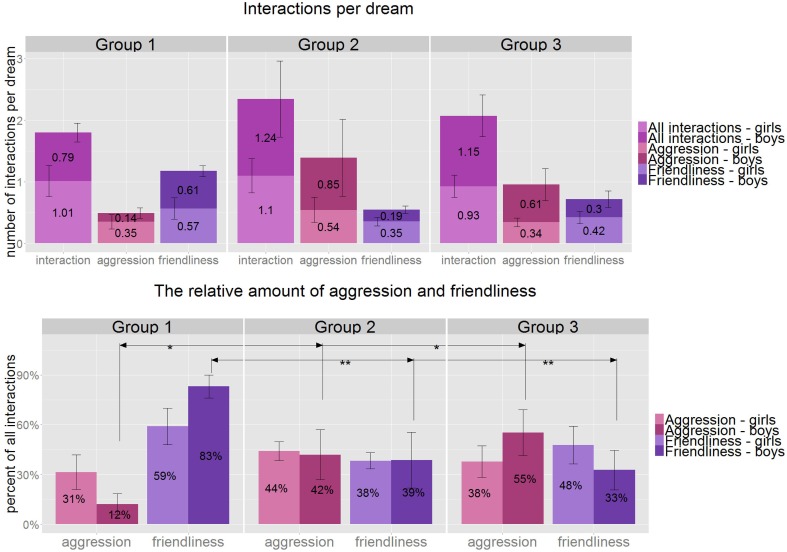
**Above:** the average number of interactions (purple), aggressive interactions (pink) and friendly interactions (blue) per dream by gender and age group. All of these variables stay relatively stable across the age groups. **Below**: The percentage of aggressive and friendly interactions in all interactions by gender and age groups. There is a significant increase in aggression from Group 1 to Group 2 and from Group 1 to Group 3, which is only significant amongst the boys and not the girls (although it stays significant without the gender split). Friendliness shows a significant decrease in the same pattern: from Group1 to 2 and from Group 1 to 3. ^*^p < 0.05, ^**^p < 0.01, Group 1: 3.8–5.5 years, Group 2: 5.51–7 years, Group 3: 7.01–8.5 years.

Neither the number of interactions per dreams nor the relative number of aggression or friendliness did show any gender related differences. However, when we paid more attention to the individual patterns of girls and boys across the age groups we discovered that both the increasing tendency of aggression and the decreasing pattern of friendliness was caused by a change only in the boys' group (Kruskal–Wallis, *H* = 5.9, *p* = 0.052 and *H* = 8.2, *p* = 0.016, respectively), while girls' relative aggression and friendliness stayed stable (Figure 4).

### Cognitions

**Figure 5:**
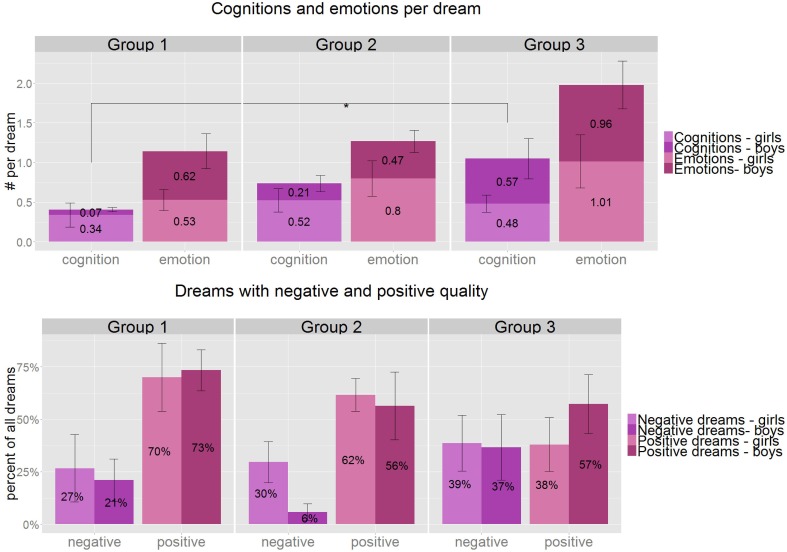
**Above:** the average number of cognitive verbs and emotions per dream by gender and age group. There is a significant increase of cognitions appearing in the dream narratives between Groups 1 and 3 (*U* = 50, *p* = 0.028, *r* = 0.42). **Below**: The ratio of dreams with negative (purple) and positive (pink) dream quality by gender and age group. There is no significant change between the age groups, although we see a tendency of increase of dreams with negative dream quality and a relative decrease of positive dreams with age. ^*^p < 0.05, Group1: 3.8–5.5 years, Group 2: 5.51–7 years, Group 3: 7.01–8.5 years.

Verbs reflecting cognitive activities were counted throughout the dream reports in order to test the parallelism of wakeful cognitive skills and dream narratives. The overall frequency of cognitive verbs in the dream reports was 0.37 and 28% of the dreams contained at least one cognitive verb. We found a significant increase of cognitions between Groups 1 and 3 (Mann–Whitney *U* = 50, *p* = 0.028, *r* = 0.42, *df* = 26) and a tendency between Groups 1 and 2 (Mann–Whitney *U* = 50.5, *p* = 0.086, *df* = 24; Figure 5).

No gender related difference was found in the number of cognitions appearing in dream reports.

Typical examples for cognitions in the dreams: “… my tooth fell into my hand and I told the teacher about it but she did not *know* where to put it…” or “… a bad person came into our house … and she pretended to be our mother … and we really *thought* she was the real mother…” (girl, 5.7 y).

### Emotions in the dreams

The assessment of emotions in the dreams was based on the self-report of the children given as an answer to the standard interview question asked by the parent. Unfortunately, this question was not evenly asked by all the parents. Here we only analyze those children's dreams whose parents reliably asked this interview question. Here our sample consists of 33 children 10 (female = 6) from Group1, 9 (female = 6) from Group2 and 14 (female = 7) from Group3, whose mean age by group does not differ significantly from the original sample.

The overall frequency of emotions appearing in dream reports is 0.85, which means almost one emotion per dream on average and the ratio of dreams with at least one emotion is 64%. Importantly, the number of emotions in dreams as well as the number of dreams with emotions are stable across the age groups and between the genders (Figure 5).

#### Emotional dream quality

More than half (59%) of the dreams were reported as positive, 27% as negative and 13.5% as neutral. Although, there is a slight increase in the number of negative quality dreams (24, 20, 38%) and a decrease in the number of positive quality dreams (72, 59, 47%) across the age groups, these remain non-significant. There was no gender related difference in affective dream quality (Figure 5).

Finally, we tested the intercorrelations of positive feelings in dreams, positive dream quality and friendly interactions in the dream reports, since they showed a similar decreasing tendency when compared to negative feelings, bad dream quality and aggressive interactions respectively. Reported positive emotions correlate significantly with positive affective quality (tau = 0.67, *p* = 6.2 × 10^−8^, *df* = 32) and negative emotions with negative affective quality of the dreams (tau = 0.69, *p* = 6.5 × 10^−8^, *df* = 32), but the relative amount of aggressive or friendly interactions did not correlate either with reported feelings nor affective dream quality.

## Summary and discussion

### Setting and data collection

In this work we put extensive emphasis on and would like to articulate the importance of methodological background in developmental dream research. We collected dreams from morning awakenings over a period of 6 weeks in a home based setting. Our aim was to create a well-controlled method, but at the same time to avoid the nighttime awakenings and the artificial environment of laboratory studies, which might affect young children's dream reports (Resnick et al., 1994; Sándor et al., 2014). Regarding the dream narratives we controlled for possible confounding factors such as parental suggestions and products of the children's waking fantasy. The 6 weeks long dream collection period provided a more representative sample of dream production than other home based studies so far (Resnick et al., 1994; Colace, 2010).

The drawback of this method is that we cannot determine the sleep stage from where the dreams were recollected upon morning awakenings, especially as the children sometimes reported dreams from various earlier phases of their sleep. However there is evidence that suggests that there is no significant difference between REM and NREM dream reports if collected close to the morning arousal (Cicogna et al., 1998). Additionally we did not control for school days vs. holidays in this study, which may influence the time of morning awakenings.

### Report rate and length

The overall median recall rate in the present study (15.5%) is similar to the cross-sectional results of Foulkes et al. (1990) where he found 20% median recall rate amongst the 5 to 8 year-olds. Pooled dream recall was 21% of the morning interviews, which is also similar to Foulkes' reports (Foulkes, 1982, 1999) on 3 to 5 year-olds (27% from REM awakening and 6% of NREM awakenings), but considerably lower than the result of the home based study of Resnick et al. (1994) revealing a 65% report rate from morning awakenings (amongst 4 to 10 year-old children, *n* = 14). However, it has to be mentioned that authors used various awakening methods during the 13 days of dream recall, and the age range of their subjects was slightly different from ours.

The overall increase in dream recall frequency (which reaches 48% of dream recalls from REM awakening and 21% of NREM awakenings in the 7 to 9 year-olds group), was not replicated in our study (Figure 1). Obviously, direct and deliberate comparison of the studies is not possible because in the present work we do not know the sleep phase that the child woke from and also the lengths of the data collection period was significantly longer in terms of consecutive mornings of dream interviews. In fact the long period of data collection had a significant effect on the dream recall frequency. This could reflect the great enthusiasm children (and parents) were involved in the task from the beginning and also could reflect the fading away of the motivation after a number of mornings beginning with the same questions.

It is intriguing that the stagnating number of dream reports across the age groups masks a peculiar gender difference: while girls relative dream recall frequency shows a clear rising tendency from the youngest to the oldest age groups, boys' report rate drops (from 46 to 25% of the total number of reported dreams). Thus, in the oldest age group we observed that girls report significantly more dreams than boys. This growing difference could be viewed as an early effect of society toward the genders (Schredl et al., 2015). The different socialization of the two genders affects their dream sharing habits resulting in different attitudes toward dreaming which then directly affect their dream recall frequency as Schredl and colleagues speculated (Schredl et al., 2015).

Regarding report length children improved from 38 words (Group 1) to 58 words (Group 2 and 3) on average. This yielded a significant difference between the first and the second group (Figure 1). Although, laboratory-based research reveals lower numbers of words (Foulkes, 1982), if we compare our findings on average report length with outcomes of the home study of Colace (2010), we see rather similar results (35 words for the 3 to 5 year-olds, and 41 for the 5 to 7 year-olds). Developmental psychologists conclude that narrative abilities are still immature in preschoolers (Pitcher and Prelinger, 1963) that is consistent with the age-related increase in dream report length found in both previous (Foulkes, 1982; Colace, 2010) and present findings. On the other hand, these results are clearly revealing that the method of dream collection can have serious impact on the formal characteristics of children's dream reports especially among young children, as we already described in our recent review of a wide range of empirical works (Sándor et al., 2014). We speculate that the nighttime awakening protocol is most distressing for the youngest children, and this effect is then observable in their dream reports in the form of shortness, infrequency and mundaneness.

### Dream characters and active self-representation

Active self-representation was relatively high in the dream reports of all age groups of our study appearing in 78% of the dreams. Although, values showed an increasing trend across the age groups, differences did not reach statistical significance (Figure 2). Other non-laboratory studies reported values ranging between 59% (Honig and Nealis, 2012) and 85% (Resnick et al., 1994) in preschoolers' dreams. These convergent findings cohere with our present results and contrasts the statement claiming the inability of young children to depict themselves as active agents in their dreams (Foulkes, 1982). It has to be noted that the active self was defined as explicit report of any movement performed by the dreamer in his dream, which might be a stricter criterion than ours. For better comparability we also calculated the ratio of dreams with self-movements and found it to be 75% amongst the preschoolers, which is still considerably higher than the 13% reported in laboratory studies (Foulkes, 1982).

In addition to the self-portrayal of the dreamer there were several other characters clearly and unambiguously mentioned in the dream reports of the 4 to 8 years-old children (3.13 characters/dream report on average, Figure 2). This value stayed relatively stable across the age groups, indicating the predominance of human characters (69% of all characters), among which family members were relatively frequent (28% of all characters). Our findings on characters in children's dream reports are comparable with adult standards of Hall and Van de Castle (1966), who reported 2.6 characters per dreams on average and 2.47 human characters per dream (95% of all characters). Other home-based studies on children's dreams reported similar results: 2.2 characters per dreams on average (2.7 for the 4 to 5 year-olds and 1.8 for the 8 to 10 year-olds), 30% of which were family members (Resnick et al., 1994). Furthermore, Honig and Nealis (2012), found that 89% of the dreams contained human characters and family members were the most common characters in the dream reports of 3 to 5 years-old children (30% of the dream reports). The adult standards indicate that 11% of all characters are family members (Hall and Van de Castle, 1966). Although, this percentage is lower than the 28% in our child sample, we also observed a trend for an age-dependent decrease of this ratio (34, 27, and 23% in groups 1, 2, and 3, respectively). Thus, our findings cohere with other non-laboratory studies and contrast the laboratory reports indicating the infrequency of human characters in the dreams of preschoolers (Foulkes, 1982).

Authors of the laboratory studies considered animal characters to be especially frequent in preschoolers' dreams and found them in 45% of the girls' and 33% of the boys' dreams (Foulkes, 1982, 1999). As a comparison 17% of the dreams contained at least one family member and “other known persons appeared even less” (Foulkes, 1982, p. 48, 1999). Additionally, a reanalysis of the laboratory results (Foulkes et al., 1990) found that 23% of all characters were animals (Domhoff, 1996) in the dreams of 5 year-olds. According to our results only 14% of the dreams contained animals and 8% (0.25 animals per dreams) of all characters were animals, which is only slightly higher than the 0.13 animals per dreams (5% of all characters) of the adult standard (Hall and Van de Castle, 1966) and very similar to the results of the Resnick-study (Resnick et al., 1994) indicating 0.19 animals per dreams corresponding to a value of 8.7% animal characters/all characters.

On the whole we did not find significant changes in the number of dream characters between the ages of 3.8 to 8.5 years. Instead we found that even 4 to 5 year-olds' dream reports are comparable to adult standards. Our home based results derived from a 6 weeks sampling procedure rather confirm earlier home and school based results. The age-dependent decrease in the number of family members in the dreams might parallel the increasing number of strangers in children's dreams (Resnick et al., 1994) reflecting the changing social exposure of the children at different ages. The high rate of dreams depicting an active self indicates that children are able to represent mental states and reflect on their own self in their dreams.

### Kinematic imagery and actions in dreams

Our findings that 86/80% of children/preschoolers report motion in their dreams confirm Honig and Nealis' (2012) results derived from preschool interviews (they found actions in 81% of the dreams). Thus, in coherence with Honig and Nealis (2012) we found that children reported motion in the majority of their dreams.

The above results on self-reported movements were further confirmed by counting explicit self-initiated actions in the dream reports. We found 4.7 actions per dream on average (3.8 for the youngest age group) and 90% of the dreams (87% in the youngest age group) contained at least one action (Figure 3).

Although, dream activities have a tendency to become more frequent with increasing age, our results prove that, even young children's dreams are highly eventful and evidently non-static. This leads us to the conclusion that children's cognitive architecture is already functional in spontaneously constructing motion imagery. This latter phenomenon contradicts Foulkes' speculations who found young children's dreams to be static and hypothesized that preschoolers are unable to imagine motion in space. In fact recent studies of children's waking spatial imagery indicate that preschool aged children have some ability to mentally represent movements and rotation, although these skills undergo significant development and show important individual differences during the preschool ages (Newcombe and Frick, 2010; Frick et al., 2013).

### Social interactions

Social interactions are present in almost every dream report and the overall aggression and friendliness per character ratio is 0.14 and 0.16, respectively. Thus, our values are higher than the aggression/character and friendliness/character ratios (0.05 and 0.06) of the laboratory dream reports in children (Foulkes et al., 1990; Domhoff, 1996). Although, the number of interactions is equally distributed among the age groups, the relative percentage of aggressive interactions does show a significant increase and the relative ratio of friendly interactions a decrease across the age groups (Figure 4). The number of dream reports with at least one aggression increases from 15% in Group 1 through 32% (Group 2) and reaches 36% amongst the 7 to 8.5 year-olds in our sample. Interestingly, in a Spanish study (where dreams were collected with the last remembered dream method) Oberst et al. (2005) found that 73% of the dreams contained aggressive interactions amongst the 7 to 8 year-olds, which gradually decreased with age until adolescence. Adolescent values were similar to the normative data of adults (45.5%). Unfortunately, Oberst did not study younger children than 7 year-olds. The age group (7–8 years), when both studies found the highest ratio of aggressive content, is the age when the children start attending school. This significant event might cause substantial challenges for children and could affect their dream contents. As regarding the absolute difference in aggressive contents of 7 to 8 year-old children's dream reports we have to mention the “last remembered dream” technique used by Oberst et al. (2005) might favor the report of emotionally salient and memorable dreams in comparison with mundane ones (Foulkes, 1999). As emotionally striking dreams often contain aggressive imagery, this could account for the observed difference in results.

On the whole we observed that a while a typical aggressive act in young children's dreams usually involves the dreamer or their close family, 7 to 8 year-olds are more likely to include a wider environment in their aggressive content. For example a typical aggressive dream from a 5.7 year-old boy: “We went to a park with father and a dinosaur attacked us…” and a 3.8 year old girl:”… I climbed onto a sofa, the cat came and put its nails into it. I kicked the cat and I kicked it again when it came back. Then it climbed onto the sofa and ate my leg.,” another example from an 8.4 year-old boy: “I dreamt that there was the third world war … and we opened the door and they were shooting on the street.”

We found the percentage of dreams with friendly interactions to be 35% in the overall sample, which is close to the average adult standard (40%). Moreover, we found that preschoolers (Group 1) have far the highest ratio of dreams with friendly interactions (45%), which is even slightly higher than that of the adult sample.

### Cognitions in the dreams

For investigating cognitive maturation we counted the number of cognitive and self-reflective verbs appearing in the dream narratives. This number increased steadily from 0.2 (Group 1) to 0.52 (Group 3) per dream. It suggests a significant age-related increase in cognitive activities in dreams (Figure 5). This finding could be explained by the general development of narrative skills involved in the dream production process, but we could also assume that it is a reflection of wakeful cognitive development modulating the dream report behavior and not dream production per se. Both hypotheses are supported by the analysis of the cognitive and reflective activities appearing in dream reports of adults: wakeful cognition parallels dreaming cognition and the emerging differences between the two would be rather quantitative than qualitative in nature (Bradley et al., 1992; Kahan et al., 1997).

Although, only 15% of the dream reports of the youngest age group contained verbs reflecting cognitive effort and metacognitive activity, this value gradually grew to 39% amongst the 7 to 8.5 year-olds. It proves that high-order cognition is present in children's dreams and it gets more prominent with increasing age. Although, we need further investigations to test how the development of this phenomenon follows waking cognitive actions and reflective abilities, baseline data could be of interest for theoretical models of reflective abilities and metacognitive functions.

### Emotions in the dreams

We found an average of 0.85 emotions per dream report which ranges from 0.72 in the youngest age group and reaches almost one emotion per dream (0.99) in the oldest (Figure 5). In fact, the average of 1.18 emotions per dream report in the adult standard sample of Hall and Van de Castle (1966) is close to the results of Group 3 (0.99) in our study. In line with the above findings the number of dreams containing at least one emotion ranges between 61% (Group 1) and 66% (Group 3), which is higher than the results of the laboratory studies [8–25%(Foulkes, 1982)], but lower than the outcomes of home (85–89%) and school based studies (79%) of Resnick et al. (1994) and Honig and Nealis (2012), respectively. Foulkes (1982) explains his scarce results of emotions in preschoolers' dreams by stating that “feeling itself is a cognitive achievement,” which he bases on the work of Schachter and Singer (1962). It is obvious that cognitive development is necessary for emotional recognition and labeling, as it was indicated in the cited work of Schachter and Singer, but cognition is not indispensable for the subjective experience of emotional events. There are several criticisms raised with respect to the cognitive appraisal theory of emotions (Zajonc, 1984). As the labeling of dreamt emotions could cause difficulties for preschoolers, we provided a list of six emotions in the dream interview. Children were explicitly asked about these feelings in their dreams. More than 60% of the dreams were labeled with at least one of the emotions by our preschooler subjects, which could have been caused by attempts to satisfy assumed parental expectations. However, a study investigating the matching of emotions to stories showed that preschoolers were highly effective in choosing the appropriate affects to the actual narrative structure (Camras and Allison, 1985).

We have to mention that only 70–75% of laboratory dream reports of adult subjects contain any emotion (Foulkes et al., 1988; Strauch and Meier, 1996; Fosse et al., 2001). Moreover, adult volunteers tend to attribute many more emotions to their non-laboratory dreams than do blind judges when they are asked to recall the emotions that accompanied the report they have written down (Merritt et al., 1994; Kahn et al., 2002). Thus, it could be that the results from self-ratings of home dream reports are due to two extrinsic factors: the demand characteristics of such a rating task, and the waking-life assumption that certain emotions would logically be present in many of the situations experienced in the dream. Although, this consideration might be relevant when interpreting our findings, it is important to note, that the striking children vs. adult difference in the laboratory dream emotions were not replicated in our home-setting investigation. In other words reporting of dream emotions in young children and adults seems to be similar when research is based on home-collected dreams. It should be mentioned that self-rating and direct questioning of emotional experiences might result in the overemphasis of emotions in dream reports, but blind judges might underrate such experiences, since they do not have direct access to them.

As regarding the emotional quality of dreams in children, our result cohere with the laboratory findings indicating the predominance of positive emotions in preschoolers' reports (Foulkes, 1982). This is consistent with the overall conclusion of those studies claiming that nightmares in both children and adults are highly overestimated because of exclusive or dominant reliance on retrospective questionnaires (Zadra and Donderi, 2000; Robert and Zadra, 2008).

On the whole we consider our method as an appropriate and efficient aid for eliciting reports on the affective aspects of dreams in children who are not comfortable with labeling emotions by themselves or think that emotions are implicitly present in the dream narrative so it is not necessary to explicitly report them (Bauer, 1976). Moreover, we conclude that emotional load is an important aspect of children's dreams which means that developmental dream research could be a valid and important extension of adult dream theories which emphasize the emotional regulatory functions of dreaming.

### Gender differences

Interestingly, we found gender differences to be relatively scarce in the present sample. The most prominent differences were those already established in the literature, namely that female subjects report dreams more frequently than males and that the majority of the characters tend to match the gender of the dreamer and this difference was present already in the preschooler's age group similarly to what Domhoff reports from the age of 5 years (Hall and Van de Castle, 1966; Domhoff, 1996). Both the increase in the relative amount of aggression and the decrease in friendliness are caused entirely by the age-effects in the dream reports of boys. Girls' relative aggression and friendliness stays stable. In contrast to previous findings (Oberst et al., 2005; Honig and Nealis, 2012), boys and girls did not differ significantly in measures of aggressive interactions at any age (Figure 4). By corroborating our finding on the gradual emergence of gender differences in dream recall frequency with the claim that gender-related attitudes are becoming accentuated with increased age (Foulkes, 1999), the dream socialization theory of Schredl et al. (2015) becomes further support. The relative age-related increase in measures of aggression in the dream reports of boys could also reflect an effect of socialization, since boys are freer to express aggression than girls. Another possible explanation of gender-related differences in aggressive interactions in dream reports could be rooted in the reported delays in the emotional maturation of boys relative to girls (Brody, 1985; Brody and Hall, 2008).

### Overall discussion

Our study considers several open questions and methodological issues in developmental dream research, reacting to many well-known and formerly non-controlled problems of the field. Our aim was to present well-controlled data from a reasonable number of subjects over an extended period of data collection in order to increase the reliability of the findings. Moreover, the tradeoff between objectivity and familiarity was handled by training the parents for using a semi-structured interview with their children. Additional controls for the reliability of dream reports were also introduced.

An important and unfortunately often neglected aspect of dream research is the indirect nature of the data; we only have access to the verbal narrative and not the dream experience itself. We should always keep in mind that the verbal and narrative abilities and memory capacity of the children may shape, affect or even limit the dream reports. Since older children and girls tend to have more advanced cognitive skills, such as verbal and memory abilities (Halpern, 2011), the possible distorting effects of gender and age could be reflected in differences of dream reports. This could be a potential confounding factor in the case of improvements in dream report length with age as well as for the tendency of girls to produce longer dream reports than boys. Besides dream report length we also found significant age-related increases in the number of self-initiated actions and cognitions in children's dream reports. There was no gender difference in these latter measures, however. These results suggest that the age-related developmental maturation of the cognitive architecture of 4 to 8 year-old children promotes the depiction of self-initiated actions and cognitive activity in their dream reports. The former finding is similar to the reports of Foulkes (1982, 1999) regarding the age-related increase in the depiction of the active self in the dream plots of children. Future studies need to differentiate the waking cognitive abilities specifically related to the appearance of self-initiated and cognitive activities in the dream reports of children.

Thus, our results support the basic concept of Foulkes' claiming that children's dream narratives follow a developmental pattern of some kind. The age-related increase in the lengths, self-initiated actions, and cognitions in the dream reports are the subject of maturation and growth during ontogenesis. At the same time, the results also suggest that even at a preschool age the level of most measures of dreams are significantly closer to adult standards than the laboratory based approach had concluded. Some researchers claim that visual imagery is highly underdeveloped in young children, preventing them to create complex dream images (Kerr, 1993; Foulkes, 1999; Kerr and Domhoff, 2004). Foulkes hypothesized that immature symbolization skills of pre-school aged children have a direct effect on the dream experience itself: “The unique properties of dreams at 3 to 5 year-olds—their static quality, their representation of salient body states rather that symbolization of social interactions, their lack of motoric self-involvement and most often of any effective form of self-representation—are dictated not by the peculiarities in the child's waking experiences but by immaturity in the child's ability to recreate such experiences symbolically” (Foulkes, 1982, p. 54). Other authors conclude that indeed young children are not only able to create images with their mind's eye, but spontaneously use mental imagery to emulate real events and boost performance of difficult or unfamiliar tasks during wakefulness (Burnett Heyes et al., 2013). Moreover, Fonagy et al. (2004) have shown that children as young as 3 years old are able to understand and engage in pretense play, which requires the simultaneous symbolic representation of the outer and inner reality. Based on these studies, as well as on our current findings we assume that even preschoolers are able to represent mental imagery such as vivid and eventful dream scenarios. On the other hand it is plausible to hypothesize that the cognitive sub-processes that support the deliberate generation, manipulation, and maintenance of mental images undergo protracted development throughout childhood and adolescence, underpinned by the maturation of executive and processing capabilities (Burnett Heyes et al., 2013) and this partial immaturity of visual imagery might influence dream production playing a role the rarity of dream recall in preschoolers.

According to the present results we conclude that although the developmental pattern is clear in some aspects of children's dreams, we also found that even preschoolers are able to represent active self-involvement, self-initiated motoric actions and kinematic imagery, various human characters and social interactions, emotional and cognitive involvement in their dream narratives. This is a significantly different picture of preschoolers dreaming than results of laboratory studies suggest, and which difference is presumably rooted in the setting and/or the method of awakening the children. Since this study is not aimed to find out the causes of the differences, further research is needed in this field.

The difficulty of investigating dream experiences in children is probably due to the inherent methodological problems of the field. Although we aimed to overcome some of these problems, like those caused by the unfamiliar research environment or incomplete arousal from sleep or suggestive questioning and confabulation (see Section Materials and Methods), several limitations and doubts have to be mentioned. Among these are the possible bias of recruiting parents who are highly interested in scientific achievements and/or motivated to get feedback about their child's development, the credibility of the dream reports, which is an untestable issue present with any dream collection method. There are efforts in the literature to address this latter problem for example one of the preschool-based studies provided opportunities (different clipboards as visual cues) for the children to dictate either a dream or a story to their teacher (Honig and Nealis, 2012). Moreover, the possible confounding factors of the environment like family status, the number of siblings, the interviewing parent's personality and the attachment quality between the parent and child which circumstances remained uncontrolled for in this study. Another limitation could be the coding system, which was aimed to integrate the advantages of two different well-used coding systems, but finally is a bit different from both of those making it difficult to find direct comparisons between studies. And finally our method of eliciting dream emotions from the children might be prone to bias from waking interpretation or social pressure. For these reasons we consider it essential for further studies on this material to start with a control for emotions by independent coders.

## Future directions

In our view the main direction of developmental dream research should be the consensual description of the characteristics of children's dreams and the clarification of the effects of methodology on the outcomes. This topic would benefit from laboratory studies of preschoolers' dreams collected upon spontaneous or scheduled morning awakenings instead of nighttime awakenings.

Since the only directly observable aspect of the dream experience is the dream report, as verbalized by the children, it would be also very important to systematically compare and analyze the similarities and differences of narratives on perceived and dreamt events.

After the descriptive analysis of children's dream narratives the next step could be the testing of the waking emotional, cognitive, and social maturation of children in connection with their dream characteristics and narrative performances.

It would be also interesting to have a closer look on the relationship between the appearance of cognitions in children's dreams and the cognitive developmental indices such as mentalization skills. We consider this point as one of special importance since the metacognitive and reflective verbs that were defined as cognitions in the children's dream reports in our current study could be reflections of the overall developmental level of mentalization skills.

As a global measure of maturation it would also be important to compare the developmental patterns of dream reports to wakeful abilities and objective electro-physiological measures of neural development such as connectivity measures of the brain electrical activity patterns. These investigations could improve our insights in the presumed relationship between dreaming and neural maturation as well as in the origin and functions of dreams in general.

### Conflict of interest statement

The authors declare that the research was conducted in the absence of any commercial or financial relationships that could be construed as a potential conflict of interest.

## Footnotes

^1^The original system of content analysis used in this work is available from the first author.
