# Supplementary material for: Content analysis of 4 to 8 year-old children's dream reports
Source: Front Psychol. 2015 Apr 30;6:534. doi: 10.3389/fpsyg.2015.00534 (PMC4415323; doi:10.3389/fpsyg.2015.00534)
Supplement: Supplementary file 1 [file Table1.DOCX]

## Supplementary material 1

## An example of dream interview carried out by the mother of a 6-year-old girl:

Mother: Did you have a dream tonight while you were sleeping?

Child: Yes.

M: Would you please tell me about it?

C: I dreamed that I was called on my cell phone and when the call ended, I somehow found myself riding a bike in the kindergarten. And then I had a great time.

M: Were you alone there or did you see anyone else?

C: The others were riding their bikes too.

M: Who were the others? ... your group- mates?

C: Yes. And do you know who called me on the phone? Papa!

M: Are you sure?

C: Yes.

M: What else do you remember?

C: I was talking to him. And I said that: “At last someone is calling me!”

M: So you talked to your dad firstly and then you were at the kindergarten riding the bike together with the others.

C: Yes.

M: Did you see these scenes as moving, like a movie or were they rather like still pictures like a photo?

C. They were moving.

M: How was this dream? Was it good, bad or neutral?

C: Good.

M: Did you have any feelings in the dream?

C: I felt good. It was good.

M: Anything else?

C: Nothing.

M: Did this good feeling stay with you when you woke up or did it go away with the dream?

C: I still feel it a little bit…
